# Adjuvant therapy of melanoma with interferon: lessons of the past decade

**DOI:** 10.1186/1479-5876-6-62

**Published:** 2008-10-27

**Authors:** Paolo A Ascierto, John M Kirkwood

**Affiliations:** 1 Unit of Medical Oncology and Innovative Therapy, Melanoma Cooperative Group, National Tumor Institute, Naples, Italy; 2 Department of Medicine, Division of Hematology/Oncology, University of Pittsburgh, USA; 3 Melanoma and Skin Cancer Program, University of Pittsburgh Cancer Institute, USA

## Abstract

The effect of interferon alpha (IFNα2) given alone or in combination has been widely explored in clinical trials over the past 30 years. Despite the number of adjuvant studies that have been conducted, controversy remains in the oncology community regarding the role of this treatment.

Recently an individual patient data (IPD) meta-analysis at longer follow-up was reported, showing a statistically significant benefit for IFN in relation to relapse-free survival, without any difference according to dosage (p = 0.2) or duration of IFN therapy (p = 0.5). Most interestingly, there was a statistically significant benefit of IFN upon overall survival (OS) that translates into an absolute benefit of at least 3% (CI 1–5%) at 5 years. Thus, both the individual trials and this meta-analysis provide evidence that adjuvant IFNα2 significantly reduces the risk of relapse and mortality of high-risk melanoma, albeit with a relatively small absolute improvement in survival in the overall population.

We have surveyed the international literature from the meta-analysis (2006) to summarize and assimilate current biological evidence that indicates a potent impact of this molecule upon the tumor microenvironment and STAT signaling, as well as the immunological polarization of the tumor tissue in vivo. In conclusion, we argue that there is a compelling rationale for new research upon IFN, especially in the adjuvant setting where the most pronounced effects of this agent have been discovered. These efforts have already shed light upon the immunological and proinflammatory predictors of therapeutic benefit from this agent – that may allow practitioners to determine which patients may benefit from IFN therapy, and approaches that may enable us to overcome resistance or enhance the efficacy of IFN. Future efforts may well build toward patient-oriented therapy based upon the knowledge of the unique molecular features of this disease and the immune system of each melanoma patient.

## Introduction

It has been more than 10 years since the pivotal trial E1684 first showed improvement in overall survival (OS) for melanoma patients treated with adjuvant high-dose interferon (HDI) [1], but controversies continue regarding the use of interferon (IFN) as adjuvant therapy in melanoma patients. In fact, despite numerous studies of adjuvant therapy, there is perhaps less consensus regarding the treatment of melanoma patients at high risk for relapse now than at any time since the FDA approval of this regimen in 1996. Parameters that may guide the consideration of adjuvant therapy, and when interferon (IFN) is considered whether it is shorter courses or lower dosages for longer intervals remain highly variable across the globe. In recent years, several reviews exploring these issues [2-23] have focused attention upon the importance of sample size and adequate maturity and power of studies, duration as opposed to dosage, the route of administration, and the relevant endpoints – whether these are relapse-free survival (RFS) or overall survival (OS). In a previous review [24] we divided oncologists into two groups: the *optimistic *ones, better known as the IFN supporters, and the *pessimistic *ones, or physicians who discount the results of IFN. For the first group, whose number of adherents has grown in recent years, HDI represents the standard therapy based on the initial ECOG and subsequent US Intergroup studies that confirmed RFS impact and in two trials, OS impact^1^, [25-27]. The latter group has stated that IFN should not be considered standard therapy for melanoma patients, since the gains in OS are relatively small, and the side effects (or cost) can not be justified in relation to these toxicities and expenses. Table 1 summarizes the most important studies of IFN-based adjuvant therapy in melanoma, with total numbers of enrolled patients.

**Table 1:** Characteristics of the main phase III adjuvant trials in high-risk melanoma patients.

**References** ***Intergroup Trial***	**AJCC Stage**	**# Total Patients enrolled**	**Arms**	**# Patients for arm**	**RFS** *P *value	**OS** *P *value
Creagan et al. (1995)^54^ *NCCTG 83–7052*	II–III	262	2	HDI 131 Control 131	0.19	0.40
Kirkwood et al. (1996)^1^ *ECOG E1684*	IIB–III	287	2	HDI 143 Control 137	0.0023	0.0237
Grob et al. (1998)^34^ *French CGM*	IIAB	499	2	LDI 253 Control 246	0.035	0.059
Pehamberger et al.(1998)^58^ *Austrian MMCG*	IIAB	311	2	LDI 154 Control 157	0.02	n.d
Kirkwood et al. (2000)^25^ *ECOG-US Intergroup E1690*	IIB–III	642	3	HDI 203 LDI 203 Control 202	0.03* 0.17**	0.744* 0.672**
Kirkwood et al. (2001)^26^ *ECOG-US Intergroup* *E1694*	IIB–III	774	2	HDI 385 GM2/QS-21 389	0.006	0.04
Cascinelli et al. (2001)^59^ WHO 16	III	444	2	LDI 218 Control 208	0.50	0.72
Cameron et al. (2001)^60^ *Scottish MG*	II–III	96	2	LDI 47 Control 49	> 0.1	> 0.2
Hancock et al. (2004)^61^ UKCCCR-MCG	IIB–III	654	2	LDI 338 Control 336	0.3	0.6
Kleeberg et al. (2004)^62^ *EORTC 18871*	II–III	423	3	UDI 240 IFNγ 244 Control 244	0.71° 0.73°°	0.72° 0.25°°
Kleeberg et al. (2004)^62^ *DKG-80*	II–III	407	4	Iscador 102 Control 102	0.12	0.31
Eggermont et al. (2005)^31^ EORTC 18952	IIB–III	1418	3	HID-IFN 565 LID-IFN 569 Control 284	0.1*	0.2*
Eggermont et al. (2008)^32^ *EORTC 18991*	III	1256	2	PEG-IFN 627 Control 629	0.011^#^ 0.107^##^	0.78
Gogas et al. (2007)^33^ *He.Co.G*	IIBC–III	364	2	HDI 1 mos 182 HDI 12 mos 182	0.94	0.51

RFS: Relapse-free survival; OS: Overall survival; HDI: high-dose interferon; ULD-IFN: Ultra-low dose interferon; HID-IFN: high-intermediate dose interferon; LID-IFN: low-intermediate dose interferon. P value refers to comparison between HDI and control groups* or LDI and control**. P value refers to comparison between LID-IFN and control and reflects the Distant Metastases Free Survival (DMFS)*. P value refers to comparison between UDI and control° or IFNγ and control°°. P value refers to Relapse-Free Survival^# ^and DMFS^##^.

After more than 20 years of research and clinical experience with IFN, it is now time to make some definitive conclusions in order to avoid eternal discussions regarding the issues of sample size, dosage, route and duration of therapy, in order to move forward in our field. For this purpose we surveyed the international literature starting with the meta-analysis published in 2006 [28] and dealing with the adjuvant treatment of high-risk melanoma, to incorporate current biological evidence regarding this molecule and its impact in vivo, so as to arrive at conclusions that may be useful for practitioners. Perhaps new evidence will serve as the most useful guidepost for further ventures in the world of adjuvant IFN.

## Meta-analysis

Wheatley et al. [19] conducted a literature-based meta-analysis of randomized trials of adjuvant interferon versus observation in patients with high-risk melanoma. The collective analysis of these 12 trials allowed the authors to conclude that relapse-free survival (RFS) was improved with IFN (HR for recurrence, 0.83; 95% CI, 0.77–0.90 [*P *< .000003]), corresponding to a 17% reduction in the risk of recurrence. There was no clear survival benefit (HR for mortality, 0.93; 95% CI, 0.85–1.02 [*P *< .1]). The authors concluded that the evidence for clinically worthwhile survival benefit is unconvincing, given a reduction in the risk of death that was ≤ 7%, that would translate to an absolute reduction in mortality of ~3% with a confidence interval that might include a reduction of 6% in mortality. That meta-analysis did not include the E1694 trial, which is the largest adjuvant trial ever conducted in the US. The data from that vaccine trial analyzed separately with data from the remaining two ECOG studies however did not yield further evidence of a survival benefit. Subgroup analyses conducted to examine dose-response relationships in this meta-analysis indicated a significant trend towards increasing RFS benefit with increasing dosage. In fact, there was evidence to support the argument that HDI is more effective than LDI with a borderline p-value of p = .02 for the correlation of RFS with dose. However, the authors concluded that there was insufficient data to determine a dose-response relationship with HDI, as opposed to a lack of efficacy with LDI, and suggested that more data was needed to conclude whether IFN-α dose is important for OS.

Pirard et al. [29] conducted another literature-based meta-analysis of nine randomized trials of IFN versus observation in order to evaluate the effect of IFN-α on relapse rate (RR) and overall survival (OS). They reached similar conclusions to Wheatley et al., but noted improvement in the recurrence rate with interferon (odds ratio 0.74; 95% CI, 0.64–0.86) without improvement in OS. Subgroup analyses showed that overall for the range of stages, HDI and LDI decreased the RR (OR = 0.71, 95% CI = 0.54–0.92, and OR = 0.76, 95% CI = 0.63–0.91, respectively), without an impact on OS.

A critical systematic review of the international literature performed by Verna et al. [30] evaluated randomized controlled trials of adjuvant treatment for high-risk melanoma patients to derive practice guidelines, including meta-analyses and reviews published between 1980 and 2004. Reported results showed that treatment with HDI consistently produced a significant improvement in RFS. Both RFS and 2-year mortality rates were significantly improved: 2-year death rates were reduced to a risk ratio of 0.85 (95% confidence interval, 0.73–0.99; *P *< .03). The authors chose this endpoint because 2-year survival may represent a meaningful benchmark for high-risk melanoma patients in terms of recurrence. The authors concluded that considering and discussing HDI is a reasonable option in appropriate patients.

Wheatley et al. [19] encouraged collaboration between groups that had performed randomized trials of adjuvant IFN in melanoma to develop an individual patient data (IPD) meta-analysis in which longer follow-up could be included, considering that some published trial reports are from several years ago, thereby increasing the number of events available for analysis and hence the reliability of the analysis. At the American Society of Clinical Oncology meeting in 2007, Wheatly et al. [28] reported the results of an IPD meta-analysis of randomized trials utilizing IFN as adjuvant therapy in melanoma patients. The main purpose of this IPD meta-analysis was to assess the totality of current evidence and to improve the assessment of IFN in the adjuvant treatment of melanoma. Despite a previous meta-analysis, the E1694 trial of IFN versus GMK vaccine was included, and the authors noted that sensitivity analysis performed excluding and including this trial made no difference in the assessment of impact upon OS. There was a statistically significant benefit for IFN for event free-survival (EFS): OR = 0.87 (CI = 0.81–0.93), but in contrast to the findings of an earlier meta-analysis by this group, no evidence was found for a difference according to dose (p = 0.2). Even more notably, there was no evidence of a difference according to duration of IFN (p = 0.5). And most interestingly, there was a statistically significant benefit of IFN upon OS from this analysis (p = 0.008): the OR for benefit was 0.90 (CI = 0.84–0.97), with no evidence of any difference according to dose (p = 0.8) or duration of IFN (p = 0.9). This proportional survival advantage translates into an absolute benefit of at least 3% (CI 1–5%) at 5 years [28]. A subgroup analysis showed that patients with ulcerated primary melanoma had an even greater benefit from IFN (EFS: OR = 0.76, OS: OR = 0.77) by comparison with those without ulceration (EFS: OR = 0.94, OS: OR = 0.98). They concluded that IPD meta-analysis provides evidence that adjuvant IFN significantly reduces the risk of relapse and improves the OS of high-risk melanoma, even if the absolute benefit is small, and not, as in this analysis, correlated with dose or duration of therapy. [28]

## Results of pending studies

Critical reading of the major international randomized trials shows that short-term relapse risk reduction with IFN appears to be independent of dosage, while durable reduction of relapse and mortality in studies followed for intervals of 7 years and longer has been documented only with the high-dose regimen tested first in E1684 [7,12,13,15,19,21,24]. The EORTC 18952 trial results [31] suggest that IFN therapy at an intermediate 2-year window of time, prevents recurrence while on treatment. Prolonged IFN therapy improved RFS in this study, although the authors concluded that this regimen could not be recommended. Since LDI has been relatively well-tolerated in comparison to HDI (grade 3–4 toxicity in about 10% vs. 70% of cases, respectively), prolonged LDI for more than 2 years was suggested as a reasonable option for melanoma patients, considering its cost-effectiveness. For these reasons, the international community has awaited mature results of the EORTC 18991 [32] trial. In fact, EORTC 18991, which compared pegylated-IFN (PEG-IFN) [induction phase of 8 weeks (6 μg/kg/week) with a maintenance phase of 5 years (3 μg/kg/week) given subcutaneously] versus control, clarified the role of duration of therapy with IFN, and provided data upon a higher dosage of PEG-IFN and an attempted longer duration (5 years) of treatment, two issues that have been discussed at length during recent years.

The EORTC 18991 trial was undertaken to test the hypothesis that prolonged exposure to IFN through the use of newer PEG-coupled forms of IFN, given subcutaneously weekly have anti-angiogenic effects in stage III melanoma patients, where the primary endpoint chosen by the EORTC was distant metastasis-free survival (DMFS), and the secondary endpoint was overall survival (OS) [32]. However, for regulatory submission it was recommended that RFS be evaluated. The results obtained in 1,256 stage III melanoma patients show no significant impact of the regimen upon DMFS, and no impact upon the secondary goal of OS [DMFS and OS rates (p = 0.107, HR = 0.88 (95% CI = 0.75–1.03) and p = 0.78, HR = 0.98 (95% CI = 0.82–1.16) respectively]; by contrast there was a significant reduction in hazard for relapse, with reduction of RFS rate [p = 0.011, HR = 0.82 (95% CI = 0.71–0.96)] at 4 years median follow-up. Subgroup analysis showed improved impact of PEG-IFN upon RFS in stage III-N1 melanoma patients and in this subset an impact was also observed upon DMFS [p = 0.016, HR = 0.73 (95% CI = 0.53–1.02) and p = 0.03, HR = 0.75 (95% CI = 0.52–1.07) respectively], although there is no evidence of an impact upon OS [p = 0.43, HR = 0.88 (95% CI = 0.58–1.33)]. Subset effects were noted for patients with primary tumor ulceration [p = 0.006, HR = 0.59 (95% CI = 0.35–0.98)] as had earlier been reported in the meta-analysis of Wheatley et al. [2007] [28]. The trial employed two phases of differing dose intensities both administered subcutaneously (and neither yet possible to correlate to the original IV induction and SC maintenance phases of the FDA-approved HDI regimen), and an initial higher-dose intensity phase of 8 weeks: while the median duration of treatment during the first phase was 8 weeks, the median duration of maintenance therapy at the lower dosage of ≤ 6 ug/kg/dose was only 12 months and only 23% of patients were treated during the 4^th ^and 5^th ^years. These last results suggest that the EORTC 18991 trial failed to clarify the role of longer-term therapy with IFN. Unfortunately, given a suggested impact in the more favorable population of N1 (IIIA, AJCC) patients, this trial is still quite early in followup – and will be best interpreted when a maturity of 5–7 years has been reached.

Gogas et al. [33] have reported another important phase III study in 2007 in a trial that compared 1 month versus 1 year of a modified dosage regimen designed to determine whether the unique aspect of the three US Cooperative group trials that have shown durable impact upon RFS may lie in the use of IV induction with HDI. In this trial the dosage of IFN differed from the classical E1684, being reduced by 25% for the induction phase, and approximately 33% for the maintenance phase (arm A: IFN 15 MIU/m^2 ^IV. for 5/7 days weekly for 4 weeks; arm B: IFN 15 MIU/m^2 ^IV for 5/7 days weekly for 4 weeks followed by maintenance dosage of 10 MIU [total rather than per m^2^, three times a week for 48 weeks]. The trial enrolled 364 high-risk melanoma patients and reached a median follow-up of 51 months. The outcome for relapse and mortality was similar between the two arms, but given the numbers of patients accrued, this allows us to conclude at the 5% significance level only that 3-year relapse-rates of arm A were not 15% higher than the shorter treatment arm B (δ = 0.15 at 3 years). An ongoing US Intergroup trial testing one month of induction therapy at the classical dosage of 20 MIU/m^2^/day for 20 doses over 4 weeks vs. observation is more than half completed, and will require a total of 1420 patients to answer the question of whether treatment has a benefit upon relapse-free survival of 7.5% or more.

## Immunological evidence

One of the unsolved questions remains – *what is the mechanism of action of IFN*? During the last 10 years we have had a number of studies that were generally underpowered, and where eligibility allowed inhomogeneous populations to be enrolled into clinical trials testing various dosages and durations of treatment. Clearly, larger trials offer more robust conclusions, and if trials demonstrate that the modality has an impact upon only some stage subsets, and not others, it may refine our application of this modality. Attention to the mechanism of action of IFN is likely to guide the improvement of this modality more than many other maneuvers. For example, one of the most interesting debates when the E1684 trial was published was whether HDI acted through a cytotoxic or immunological mechanism. At that time many oncologists leaned toward a cytotoxic mechanism of action rather than an immunological mechanism. Only the trial of the French Group [34] provided evidence for an immunological mechanism from their clinical findings. In fact, after the publication of the results of LDI treatment in low-intermediate-risk stage II melanoma patients, they demonstrated the existence of a subset of responsive patients [35] defined on the basis of elevated white blood cell (WBC) counts where more prolonged RFS was obtained. In the last few years several important immunological findings have added strong support for this hypothesis that the mechanism of action of IFN is immunomodulatory.

Moschos et al. [36] reported data from a neoadjuvant treatment study with HDI given according to the induction phase of the E1684 trial: after induction treatment in this study 11/20 (55%) stage IIIB melanoma patients showed objective regression of palpable regional lymph node disease, and 10/20 (50%) patients were disease free after a median follow-up of 18.5 months. An important immunological finding regarding response to HDI treatment was that the number of mononuclear lymphocytes and dendritic cells were increased in the tumor tissue at 4 weeks of treatment among responders. This correlation of response with increased tumor-infiltrating CD3^+ ^and CD11c^+ ^cells, and decreased CD83^+ ^cells suggests an indirect immunomodulatory mechanism of action for this therapy [36].

Additional strong evidence for an indirect immunomodulatory mechanism of action has come from the Hellenic Oncology Group trial of Gogas et al. [37], which showed that the development of clinical and serological manifestations of autoimmunity, including autoantibodies to and clinical manifestations of autoimmunity in melanoma patients treated with HDI (26% of the total), correlates with a better RFS and OS. In fact, the Hellenic Group found only 2 deaths in 52 melanoma patients with serologic or clinical evidence of the development of autoimmunity during treatment, while there were 80 deaths among 148 patients without such evidence of autoimmunity (p < 0.001).

This phenomenon has been further explored by the Eastern Cooperative Oncology Group in a study reported by Stuckert et al. [38] in 2007: a correlation was shown between the development of autoantibodies among HDI-treated patients, and improvement of RFS and OS – but in this retrospective study only serological and not clinical manifestations were possible to evaluate. These data showed a strong trend (p=.06) for correlation of the serological development of autoantibodies during HDI and melanoma relapse and mortality – extending the work of Gogas et al., demonstrating clinical benefit with immunomodulation and induction of autoimmunity. The induction of autoantibodies may be a useful surrogate marker for monitoring the efficacy of IFN therapy.

The association between a better outcome and the appearance of autoimmune phenomena was previously demonstrated in early studies of IL-2 where thyroid autoimmune responses were shown to be strong correlates of therapeutic benefit in advanced disease, and in more recent studies utilizing anti-cytotoxic T-lymphocyte antigen 4 (CTLA-4) antibodies that act through releasing inhibitory functions mediated by this molecule in T cells [39-42]. CD4^+ ^T lymphocytes that express high levels of CD25 on their surface and the specific marker FoxP3, have shown suppressive functions upon T cells reactive with self antigens. The possibility that the Treg cells could influence the clinical outcome of cancer patients has been hypothesized on the basis of their increased number in many cancers [43-48].

**Figure 1:**
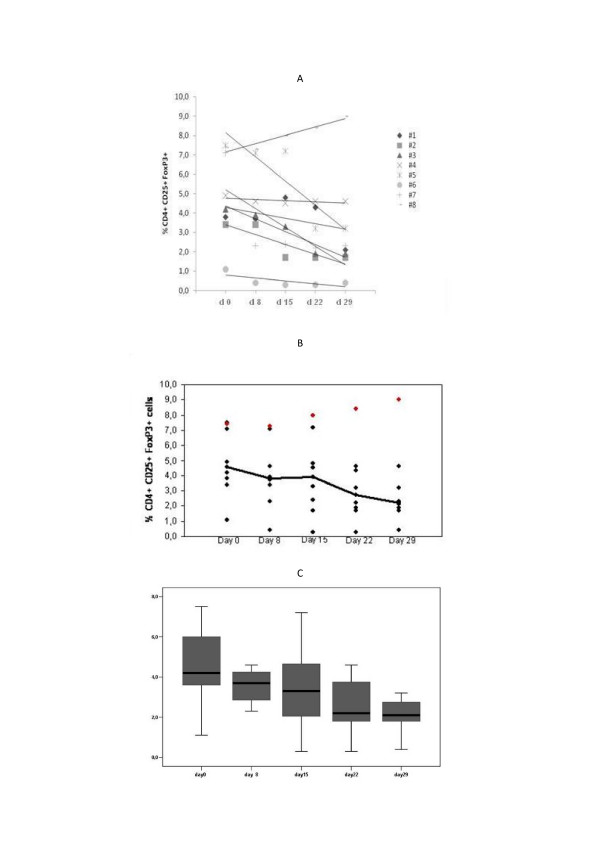
**A-B-C.** Levels of circulating Treg cells (CD4+CD25+FoxP3+) in the blood of melanoma patients during the four weeks of HDI IV induction therapy. The peripheral blood assays have been performed at the start of each week of treatment (Day 0, 8, 15, 22) and after the last week (on Day 29). (A) Trend in a single patient; (B) trend of the average value of Treg cells during HDI IV treatment; (C) Boxplot summarizing the data observed in the cohort of patients during the initial 4 weeks of treatment.

Viguier et al. [49] described increased numbers of Tregs in peripheral blood (PB) of melanoma patients and their presence in lymph nodes containing metastatic disease, capable of inhibiting the effector functions of the immune response in situ. Cesana et al. [50] reported increased basal levels of Treg in PB of melanoma and renal cell carcinoma (RCC) compared to healthy donors. Our preliminary results [51] support a possible role of HDI in relation to Treg, decreasing their number in PB with the consequent possibility of potentiation of immune responses. In fact, among 8 consecutive patients treated with HDI as a neoadjuvant or adjuvant therapy, we tested on days 0, 8, 15, 22 and 29 (after the HDI induction phase iv) the level of Treg cells in the PBMC. Our findings showed that circulating Treg levels decreased in 7 of the 8 patients (87.5%) with a median value for the drop in reduction in the circulating fraction of Treg that was 1.7% (range 0.3–4,8%) (Figure 1). Moreover, in the only patient in which we did not observe a decrease of Treg, HDI treatment was discontinued after 2 weeks for grade 3 hepatotoxicity. This provides further evidence to support the concept of an indirect mechanism of immunomodulatory action for HDI. There is a large need for further studies that correlate clinical outcome and changes in Treg before reaching any conclusions.

Molecular correlates of action for IFN would be of great use, and several candidates exist in the JAK-STAT pathway through which IFN signaling occurs. The Janus-activated kinase (JAK)/signal transducers and activators of transcription (STAT) pathway of IFN signaling are important for immunoregulation and tumor progression. Wang et al. [52] reported results in the setting of a prospective neoadjuvant trial of HDI [36] demonstrating the reciprocal effects of HDI upon STAT1 and STAT3, which appear to operate jointly as mediators of IFN effects. It has been postulated that these may be best assessed in the balance of pSTAT1 and pSTAT3. STAT1 plays a prominent role in the effector immune response, whereas STAT3 is implicated in tumor progression and down-regulation of the response to type I IFNs. HDI was found to up-regulate pSTAT1, whereas it down-regulates pSTAT3 and total STAT3 levels in both tumor cells and lymphocytes. Higher pSTAT1/pSTAT3 ratios in tumor cells pretreatment were associated with longer overall survival (P = 0.032). The pSTAT1/pSTAT3 ratios were augmented by HDI both in melanoma cells (P = 0.005) and in lymphocytes (P = 0.022). Of the immunologic mediators and markers tested, only TAP2 was augmented by HDI. Thus, Wang et al. demonstrated that HDI significantly modulates the balance of STAT1/STAT3 in tumor cells and host lymphocytes, leading to up-regulation of TAP2 and augmented host antitumor response. Moreover, the pSTAT1/pSTAT3 ratio in tumor cells at baseline could serve as a predictor of clinical outcome, and the modulation of this ratio could serve as a predictor of therapeutic effect.

Yurkovetsky et al. [53] utilized a multiplexed cytokine assay in order to analyze differences in serum concentrations of 29 different cytokines, and angiogenic and growth factors in melanoma patients and healthy controls. For this purpose serum samples were analyzed from 179 melanoma patients before HDI or vaccine adjuvant treatment and from 378 healthy controls. A statistically significant increase in concentrations of 15 biomarker proteins (IL-1α, IL-1β, IL-6, IL-8, IL-12p40, IL-13, GCS-F, MCP-1, MIP-1α, MIP-1b, IFNα, TNFα, EGF, VEGF, and TNFRII) was found in the sera of melanoma patients compared with age- and sex-matched healthy controls (P < 0.05–P < 0.001). These data showed that melanoma patients have a significantly different pattern of expression for multiple serum cytokines compared with healthy individuals. Moreover, HDI therapy induced significant changes in the serum concentrations of multiple cytokines. HDI therapy decreased levels of angiogenic and growth factors (VEGF, EGF, HGF), whereas expression of IP-10, IFN-α, MCP-1, IL-12p40, soluble TNFR-I, TNFR-II, and IL-2R were significantly increased in the serum evaluated 3 months post initiation of HDI treatment. These changes observed 3 months after HDI treatment did not correlate with outcome (treatment benefit) but it remains unclear whether earlier or later changes in cytokine concentrations might correlate with RFS. Of great interest, these data from the University of Pittsburgh show that pretreatment levels of the proinflammatory cytokines IL-1α, IL-1β, IL-6, TNFα, and the chemokines MIP-1α and MIP-1β were significantly higher in the serum of patients who were treated with HDI and had RFS longer than 5 years, compared with those who relapsed earlier. No such correlation existed between these pro-inflammatory cytokines and the outcome of patients treated with the GMK vaccine. In fact, melanoma patients with the highest levels of these cytokines had the highest rates of RFS at intervals of > 1 and > 5 years, whereas patients with the lowest levels of these cytokines tended to have RFS of < 1 year. It thus appears that the baseline cytokine milieu of the patient prior to treatment with HDI may predict the susceptibility to benefit from HDI.

A final recent study upon STAT1 and T cell signaling in the blood lymphocytes of patients with melanoma is noteworthy, and may allow us to understand the treatment benefit of HDI, that has not been seen with other regimens of IFN that do not achieve high circulating levels of IFN in the blood. The group of Lee et al. [54] have studied T cell signaling defects known to be associated with advanced melanoma, and that provide some of the impetus to consider the evaluation of new therapies in patients who do not have such far-advanced disease (as in the adjuvant setting). These investigators have used phospho-flow analyses for STAT1 phosphorylation to document a surprisingly high frequency of T cell signaling defects in the PB lymphocytes of patients with advanced melanoma (~30%). Normalization of this defect was found by *in vitro *exposure of the PB lymphocytes of these patients to high concentrations of IFN, such as would be expected to be achieved by the administration of 20 MU/m^2 ^during induction therapy using the classical regimen of HDI, but not by exposure to lower concentrations.

## Conclusion

After the most recent meta-analysis and the reports of the latest results of ongoing clinical trials testing new variations on adjuvant treatment for high-risk patients, what more do we know and what can we conclude? Recent announcements regarding the negative results for the EORTC 18961 trial (unpublished) that compared GMK vaccination with observation [OS worse with GMK (p < 0.02)], indicate that there may be less certainty in regard to the results of E1694 than previously. We, however, are strongly convinced of the contrary!

First of all, regarding the statistical investigations we have affirmed [24] that "transitive properties" do not apply for medical trials. It is a fundamental mistake to consider GMK vaccination with Tay Sachs Brain derived GM2 and Bovine or Rabbit Brain derived GM2 equivalents to synthetic GM2 as performed in the EORTC 18961 trial. In fact, in E1694 bovine brain or rabbit brain-derived GM2 has not formally been established to be immunologically equivalent to the synthetic GM2 utilized in the EORTC 18961. Comparisons of the data observed for these fundamentally different kinds of GM2 vaccinations are as different as the Salk formalinized Polio virus vaccine and the Sabin live vaccine. So the murky nature of the vaccination studies with GM2 should not compromise the 25 years of incremental understanding that has emerged in relation to IFN, and the spate of recent biological findings that have clarified the role of IFN in the adjuvant setting.

The issue of IFN treatment duration has been addressed in the recent Wheatley report [28]. It was apparent that adjuvant IFN significantly reduces the risk of relapse and improves OS (even if the absolute benefit of the survival increment is relatively small) and additionally there was no evidence that such a benefit is duration-dependent.

The EORTC 18991 trial failed in its original stated purpose, which was to evaluate the importance of prolonged durations of treatment to 5 years, on the basis of the availability of a new formulation of PEG-IFN. In fact, the median duration of treatment was only 14.9 months – barely 3 months past the 12 month E1684 regimen that 90% of non-relapsing patients in E1694 received. Ultimately, only 23% of stage III melanoma patients were treated for 4–5 years, so this trial does not permit any conclusions regarding the impact of longer durations of treatment. Moreover, it is not possible to make any comparison between HDI and PEG-IFN because, IFNα2b (used for the trials of HDI) and PEG-IFN are two different drugs administered by very different routes (IV and SC. respectively) for which there are few rigorous data based upon careful studies comparing the two of these agents for any of the relevant immunological and anti-tumor endpoints.

Secondly, in Table 2 all of the recent immunological findings correlated with HDI are summarized. It represents the first time in the history of adjuvant IFN therapy that we have strong evidence that HDI works though indirect immunological mechanisms. These data in support of the new formulation of PEG-IFN are still quite incomplete. These findings relate in general to the effects of the induction phase of HDI suggesting a critical role of the pharmacokinetics of HDI given IV. The induction phase of HDI administered according to E1684 [1] distinguished this regimen from the Mayo NCCTG regimen of 3 months intramuscularly (i.m.) with high-dose IFN as reported by Creagan (IFN-α2a 20 MU/m^2 ^i.m. tiw × 3 months) [55] which failed to alter disease outcome. While this regimen has been considered similar to the E1684 IV. induction phase, it has never been shown to achieve the blood levels of > 10,000 u/ml that have been associated with E1684 IV dosing. These findings stress the importance of the IV. route of administration and add a caveat regarding the difficulties in attempting to make comparisons between ECOG HDI and the EORTC PEG-IFN regimen, which must be considered a challenge for the future.

**Table 2:** Recent evidence for indirect immunomodulatory mechanisms of HDI

Increase in Tumor Infiltrating cells^36^
Development of autoantibodies and clinical manifestations of autoimmunity (~30%)^37,38^
Decrease in Circulating Treg cells^51^
Modulation of the STAT1/STAT3 balance in tumor cells and host lymphocytes^52^
Change in serum cytokine concentrations^53^
Normalization of T cell STAT 1 signaling defects in peripheral blood lymphocytes^54^

The Hellenic Cooperative Group study [33] has utilized a further variation upon the E1684 regimen, to compare 1 month and 12 months of treatment, and the lack of differences between the results of IV treatment with 75% of the IV induction dosage stipulated in E1684, and this induction, added to a maintenance regimen that gave 10 MIU per dose rather than 10 MIU/M2 for 11 months, supports the hypothesis that the IV induction phase of treatment is of paramount importance. The ongoing E1697 trial (comparing 1 month HDI versus observation) and the Italian Melanoma Inter-group trial (IMI – Mel.A) [56] (which compares intensified IV HDI versus the E1684 schedule) as well as the German-Austrian-Swiss DeCOG trial testing repetitive induction vs. the E1684 schedule) will give us additional information.

In Table 3 we summarize the known absolute benefit at 5 years for adjuvant therapy for several common cancers. The recent meta-analysis by Wheatley et al. [28] for a multitude of regimens, most of which have never been suggested to induce durable remission or prolonged survival, have in aggregate shown an absolute benefit for survival at 5 years of ~3% (with CI from 1–5%). Classical chemotherapy for cancers reported in Table 3 shows an absolute benefit at 5 years that ranges from 4% to 9% with toxicity that is not trivial (i.e. lung and ovarian cancer).

**Table 3:** Absolute benefit at 5 years of the most important adjuvant treatments for cancer

Adjuvant Regimen	Type of Cancer	% Absolute Benefit at 5-years
IFN vs none^28^	Melanoma	> 3.0
CMF like CT vs none^63^	Breast	4.7
Anthracycline based CT vs CMF like CT^63^	Breast	3.3
FolFox vs none^64^	Colo-rectal	5.9
Platinum-based CT vs none^65^	Lung	4.1
Platinum-based CT vs none^66,67^	Ovarian	7–9

These considerations together with recent findings that illuminate the immunological nature of the therapeutic mechanism of HDI suggest that we ought not to change our attitude about the role of adjuvant high-dose IFN in high-risk melanoma patients. It is time to deploy the therapy we have had for more than a decade, and to make individual (patient oriented) conclusions in regard to the benefit of HDI. IFN works and gives an absolute survival benefit at 5 years that may be as much as 5%. This benefit is not far from the results of uncontested therapies for other kinds of cancer. Moreover, if we can confirm the role of the induction phase of high-dose IV IFN administered during the first one month of the effective schedule, physicians could offer patients an effective treatment with very manageable and short term toxicity that would compare favorably with other adjuvant regimens for unrelated solid tumors.

Our efforts should now be focused upon determining the scientific basis of action for this modality, and those patients who benefit the most from IFN therapy, as well as how to overcome resistance to further enhance efficacy of IFN. Specifically, it is time to test new combinations with HDI in the advanced and adjuvant disease settings, starting with recent immunological findings. This will include use of neoadjuvant approaches, or the more intelligent evaluation of sentinel nodes in relation to the determinants of therapeutic benefit for IFN; second is to increase our knowledge regarding the critical biological determinants in every adjuvant trial, such as the induction of autoantibodies, and the definition of immunogenetic factors that can predict the susceptibility to autoimmunity. The real objective of future efforts will ultimately be to build a scientific basis for individualized therapy based upon the knowledge of the unique molecular features and immune system of each melanoma patient [57]. What to do in the mean time? We suggest that HDI administered in the conventional E1684 trial schedule is the most reasonable approach, and this clearly can be modified according to toxicity, and at least should include the initial component of IV induction along with careful assessment of the molecular and immunological aspects that may inform future development and refinements of this regimen.

## Competing interests

JMK is as a consultant for Eleos Inc, serves on the Speakers' Bureau for Schering Plough Corp and receives research grant support from Pfizer, Bristol Myers Squibb, MedImmune, GlaxoSmithKline and AstraZeneca.

## Authors' contributions

JMK and PA both 1) made intellectual contributions and participated in the acquisition, analysis and interpretation of data; 2) have been involved in drafting the manuscript; and 3) have given final approval of the version to be published.
